# A Collaborative Application for Assisting the Management of Household Plastic Waste through Smart Bins: A Case of Study in the Philippines

**DOI:** 10.3390/s21134534

**Published:** 2021-07-01

**Authors:** Navjot Sidhu, Alberto Pons-Buttazzo, Andrés Muñoz, Fernando Terroso-Saenz

**Affiliations:** Polytechnic School, Catholic University of Murcia (UCAM), 30107 Murcia, Spain; nsidhu@alu.ucam.edu (N.S.); apons2@alu.ucam.edu (A.P.-B.); fterroso@ucam.edu (F.T.-S.)

**Keywords:** ambient assisted living, quality of life, smart bin, plastic-waste management, route-planning algorithm

## Abstract

The management and collection of household waste often represents a demanding task for elderly or impaired people. In particular, the increasing generation of plastic waste at home may pose a problem for these groups, as this type of waste accumulates very rapidly and occupies a considerable amount of space. This paper proposes a collaborative infrastructure to monitor household plastic waste. It consists of simple smart bins using a weight scale and a smart application that forecasts the amount of plastic generated for each bin at different time horizons out of the data provided by the smart bins. The application generates optimal routes for the waste-pickers collaborating in the system through a route-planning algorithm. This algorithm takes into account the predicted amount of plastic of each bin and the waste-picker’s location and means of transport. This proposal has been evaluated by means of a simulated scenario in Quezon City, Philippines, where severe problems with plastic waste have been identified. A set of 176 experiments have been performed to collect data that allow representing different user behaviors when generating plastic waste. The results show that our proposal enables waste-pickers to collect more than the 80% of the household plastic-waste bins before they are completely full.

## 1. Introduction

Exposure to plastic waste has been evidently increasing globally with almost every industry, corporation and community relying on plastics as they have become a commodity to the citizens [1]. With the United Nations announcing plastic pollution as a global crisis, many countries have banned single-use plastics and started to urge people to help minimize their plastic usage. Furthermore, many manufacturers and producers have started changing their entire production industry to help in their own ways to reduce plastics [2,3]. In this context, an efficient collection of plastic waste is being perceived as a fundamental public service [4]. The existing solutions and policies for plastic-waste management differ per country. However, most policies are strictly based on traditional waste collection methods with fixed schedules [5]. Intelligent techniques integrated to the prevailing system could be one of the solutions to make this collection more efficient. Some studies focus on digitization of solid waste bin maps while trying to minimize the route length of the collecting vehicles [6]. Additionally, current studies rely on the use of devices such as weight sensors to measure the bin levels of a municipality in real time [7].

Despite these efforts, there are still situations in which due to no collection service, inadequate vehicle routing or insufficient funds, some communities find it difficult to have an accessible garbage collection. As stated in [8], there are many cities in developing countries that are having issues with systematically and sufficiently providing garbage collection services. To worsen this situation, due to the COVID-19 pandemic, plastic pollution has visibly increased with the production of face masks, face shields and personal protective equipment (PPE) kits to protect against the transmission of the virus while lockdowns and quarantine restrictions are also applied in some countries [9,10].

As a result of these situations, we have identified a necessity for managing the increasing household plastic waste, especially for the elderly, disabled people or people in a quarantine situation, who may find themselves in a difficulty in accessing to the plastic-waste bins at the street. Thus, the aim of this work is to develop an intelligent collaborative system to predict the state of household plastic bins to optimize the route of residential garbage collectors or waste-pickers [11,12].

The contributions of this paper are three-fold. First, we propose a simple smart bin using a weight scale sensor to monitor household plastic waste. Secondly, we design a collaborative application for managing the collection of household plastic waste focused on communities with special needs in this regard, such as the elderly, people with reduced mobility and COVID-19 affected residents. Lastly, we define an algorithm for forecasting the generation of household plastic waste to optimize collection routes for residential garbage collectors.

The rest of the paper is structured as follows. Section 2 provides an overview about current approaches for intelligent waste management. Section 3 is devoted to explaining in detail the proposed framework whereas Section 4 shows the main results from a simulation scenario in the Philippines. Section 5 discusses the findings and limitations of this work. Finally, the main conclusions and the future work are summarized in Section 6.

## 2. Related Work

This section starts reviewing different types of sensors for waste management. Then, some of the techniques for predicting waste generation are studied in Section 2.2. Finally, a review of algorithms for optimizing route planning is given in Section 2.3.

### 2.1. Sensors and Social Data for Waste Collection

The integration of Information and Communication Technology (ICT) solutions with urban development planning is one of the paramount points for the realization of smart cities. This growing technology can be used to improve the quality and efficiency of services in a city. Indeed, the concept of Internet of Things (IoT) fosters the connection and transmission of data among any type of device in a city, allowing citizens and organizations to exchange these data and create collaborative services on top of these IoT systems.

An IoT solution that made use of Radio-Frequency identification (RFID) and ultrasonic sensors to monitor municipal waste bin levels was presented in [13]. RFID readers were also placed in the collection vehicles for a faster transmission of data and efficient collection of waste.

Another similar study used ultrasound sensors in waste bins for a more efficient collection of waste. These sensors measured three distances in a bin, namely the size of the bin (i.e., the total capacity), the distance from the bottom of the bin until the filled level, and the distance of the empty part or the unfilled part of the bin. This information was transmitted to a database which scheduled the trips to collect waste and informed the collecting vehicles using an application [14].

Other types of sensors can also be used for bin level measurement. For example, in a study in [15], a load sensor was used for measuring the weight of the waste in the bin, while gas sensors were used for detecting harmful gases. Additionally, an infrared sensor was used to detect the fill level of the bin. Other contextual factors such as temperature and humidity levels were determined as well. All these data were sent to a server in the cloud and processed to show the amount of garbage in each bin and its location in an app. As stated in [16], it is possible to improve waste collection even by means of a low-cost device such as a digital scale which can track the weight inside the bin and automatically send a message to a vehicle near the area when reaching a specific threshold.

Apart from the physical or *hard* sensors reviewed above, the crowdsensing paradigm has demonstrated to be a reliable alternative to collect data for waste management. In a smart city context, crowdsensing helps infer activities and relationship of citizens as data producers [17]. In mobile crowdsensing, there are two types of sensing: participatory, in which a user agrees and decides which applications or websites will be sensing, and opportunistic, in which applications run in the background without the prior involvement of the user [18]. Crowdsensing can be integrated into gamification techniques such as presented in [19], where a game is designed to study how to match trash bins with types of waste can give points as an effective way of recycling, while producing data about the actual use of the recycling bins.

The application proposed in this work makes use of a scale sensor to measure the weight of the plastic-waste bins at the end-users’ houses. Therefore, the solution does not depend on a large and complex IoT infrastructure but on a cost-effective sensor to ease the feasibility of the proposal. Moreover, it may foster the collaboration among citizens by connecting people with different difficulties in taking out the plastic waste with waste-picker volunteers or residential garbage collectors.

### 2.2. Forecasting Techniques in Waste Management

Several studies (see for example [20,21,22]) show that the citizen’s behavior towards the disposal of resources depends on different factors such as age, gender and economic level, among others, proving also that there is a notable relationship between social norms and waste-management behaviors.

There have been efforts in the literature to predict this behavior through machine learning techniques. Thus, in Ontario (Canada), waste generated over 12 years and census data from 2001, 2006 and 2011 were filtered, extracted and extrapolated to create municipal solid waste datasets and socio-economic variable datasets. These datasets were combined and correlated with identify socio-economic parameters, such as demographics, income, education, employment and household characteristics, to be able to make predictive models for solid waste generation through artificial neural networks and decision tree algorithms [23]. In a study in [24], machine learning techniques were used to forecast the fill level of the bins and to cluster data to differentiate between biodegradable and non-biodegradable waste. Sensors were installed both in household bins and street bins with the aim of providing a more efficient garbage collection.

In the present work, an important module of the proposed application includes a forecasting mechanism to predict the waste-related behavior of the end-users. This technique relies on the hypothesis that such a behavior is reflected by the weight variations of the plastic waste allocated inside each target bin.

### 2.3. Route Planning in Waste Management

Establishing an optimal route for stakeholders to collect waste efficiently and within their range is also a substantial step for an intelligent waste management. Several studies that focus on optimized waste transportation can be noted under the Traveling Salesman Problem (TSP), which takes into account the number of waste bins and routes in three different cases [25], namely:

1. single station—single route
2. single station—multiple routes
3. multiple stations—multiple routes

Furthermore, these studies suggest multiple solutions not only based on bins and routes, but also based on the type of waste. Recyclable waste could be taken from a municipal bin using a single station with multiple routes, while non-recyclable waste could be collected by a round trip which collects and dumps when the bin is full. Lastly, routes for harmful waste could be calculated considering the time and distance of each route.

In another route-planning technique proposed in [26], it was considered a garbage collection system through an app and an alert notification technique based on the status of containers and vehicles along with citizen and social media reports. The proposal integrated an artificial transportation system, composed of sensors such as CCTV systems, cellphone data and social media data, among others, with a real transportation system using built-in GPS in trucks. The latter system is informed by the artificial system with real-time data about the fill level of bins, and therefore the collectors can either go to the bins recommended by the artificial system or follow the fixed routes allocated to the trucks. A similar study [27] highlighted the use of mathematical calculations and statistical analysis to determine the optimal distance between bins throughout the city and the best route according to the distances between trucks and bins. The bins’ locations were defined through a GIS (Geographic Information System) network analysis to optimize the planned routes.

In this context, our solution also proposes a route-planning mechanism for waste collection. However, unlike the aforementioned approaches, this collection follows a collaborative approach where a set of waste-pickers or residential garbage collectors oversee collecting the plastic waste from the household bins by means of ad-hoc routes. These routes take into account not only the current state of the household bins but the predicted one along with some contextual elements such as the collectors’ means of transport.

## 3. Materials and Methods

This section explains the key elements and techniques of our proposal.

### 3.1. Architecture Framework

**Figure 1:**
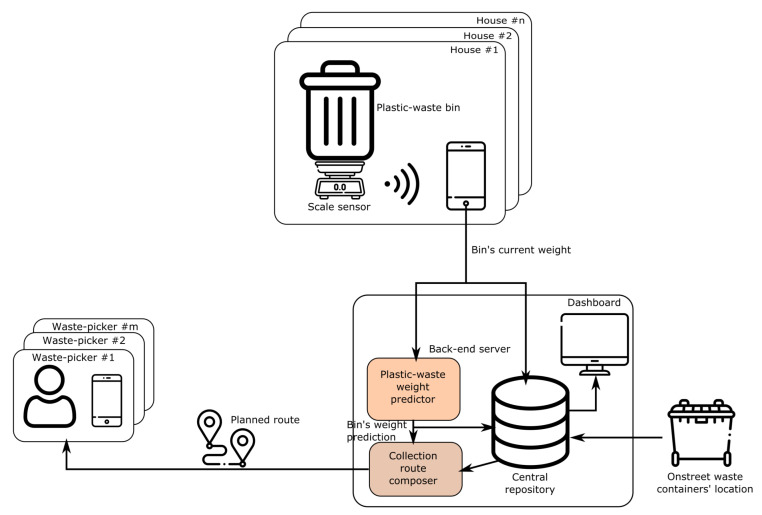
Overall architecture of the smart-collaborative waste-management system.

Figure 1 depicts the general architecture of this proposal. First, the system captures the current weight of the plastic-waste bins from a set of monitored houses by means of a scale sensor. Next, these weight data are sent to a back-end server. It comprises a dashboard to visualize all the collected sensor data. Moreover, it also includes a predictor module to forecast the future weights of all the monitored bins in the short term. Finally, such predictions are used to plan customized routes for the waste-pickers registered in the system so that they can collect the plastic waste at each monitored home in a proactive manner. To do so, both the location of the on-street plastic containers or dumps and the waste-picker’s means of transport are also considered to optimize the distances covered by these pickers.

### 3.2. Smart Bin

**Figure 2:**
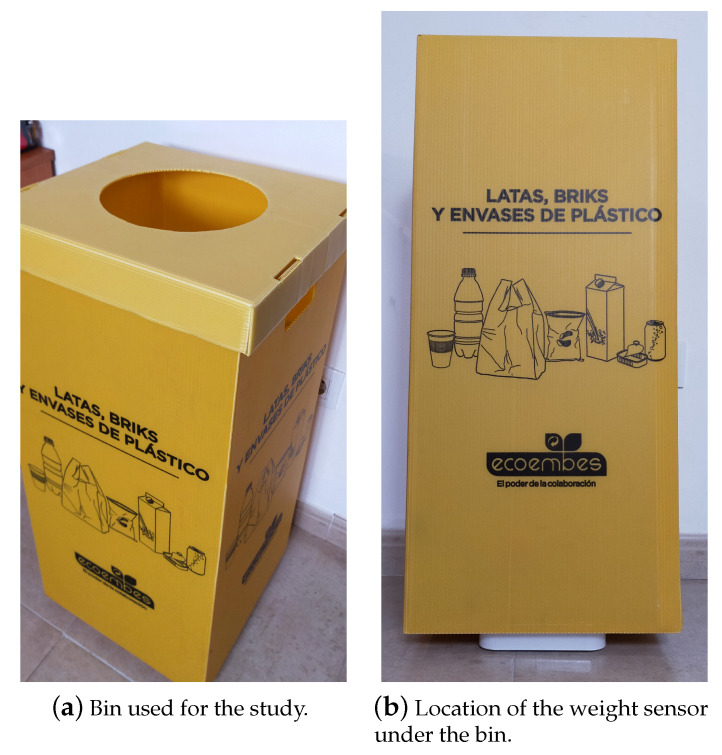
Household plastic-waste collection. The label in the bin reads “Cans, cartons and plastic packaging” in Spanish.

A plastic-waste bin with the dimensions of 75 × 34 × 36 cm was used in this study (see Figure 2a). These dimensions allowed to place plastic waste of different sizes, forms and weights into the bin. In this way, it was possible to use this bin in a large range of scenarios.

**Figure 3:**
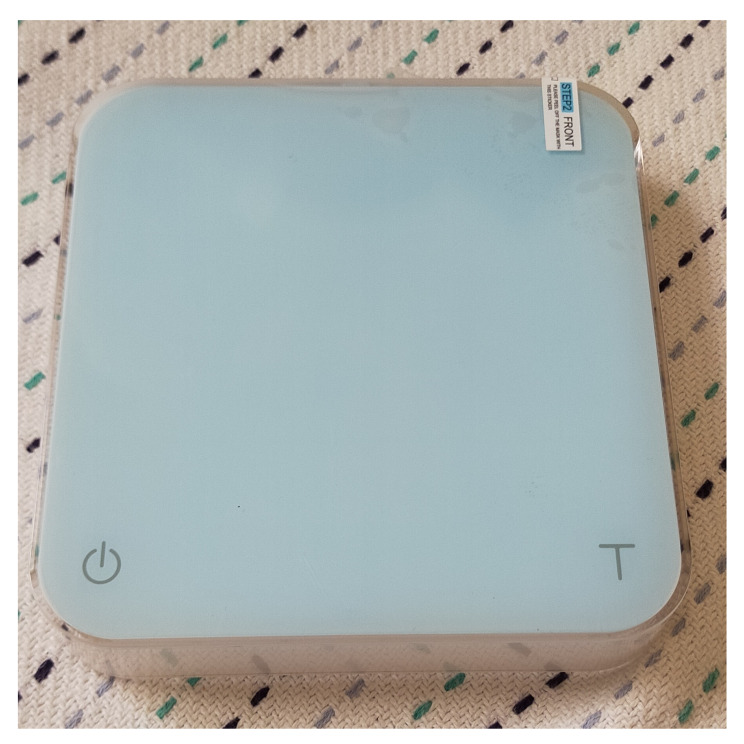
Acaia Pearl weight sensor.

The content of the bin is measured by means of a weight sensor (see Figure 2b). Since the plastic items placed inside the bin might have a very low weight, it was important to use an accurate sensor able to detect this kind of objects in the container. For that reason, we opted for a coffee brew scale. This type of weighting scale has a very high sensitivity. In particular, we made use of the Acaia Pearl model (see Figure 3), an affordable weighting scale with maximum capacity of 2000 g and a readability of 0.1 g. (https://acaia.co/collections/coffee-scales/products/pearl?variant=2433774125079 (accessed on 24 May 2021)). Furthermore, it includes Bluetooth connectivity that allows real-time transferring of the bin’s weight to a custom application installed in a mobile device (Brewmaster application: acaia.co/pages/apps).

### 3.3. Data Collection

Given the bin and the weight sensor described in the previous section, we undertook a palette of different experiments. These experiments were performed in the researchers’ environment which included their households and workplaces along with the people they were living with and their co-workers within the age group of 21–50 years old. Different types of households ranging from one to five-persons in a student housing, a workplace and a family house were explored (see Table 1). The researchers used these data to simulate the behavior assessed with the target clusters as explained in Section 4.4. In each experiment, we put the weight sensor under the bin as shown in Figure 2b. Then, we connected the sensor via Bluetooth to a mobile device to collect the weight of the bin at each moment. Next, the bin was just used normally to put plastic waste in it. Thus, we performed several experiments involving different types and sizes of plastics such as plastic bottles, cans, plastic bricks and bags. Through these experiments, different behavioral clusters related to plastic-waste generation were extracted as explained in Section 4.4. Finally, at the end of each experiment, all the timestamped measurements transmitted by the sensor were stored in a file repository (see Table 2 for an example) by the mobile device. For each experiment we stored the following parameters:

1. starting date and hour,
2. time length of the experiment,
3. the total weight of the bin at the end of the experiment, and
4. the evolution of the weight captured by the sensor during the whole experiment.

### 3.4. Data Cleaning

Due to the high sensitivity of the scale sensor, it was able to capture any minimum weight fluctuation in the bin. Even though this allowed us to perceive any plastic item placed into the bin, a side effect was that this also generated rather noisy data sequences. As a result, a data curation process was performed over the collected data. The goal was to keep only the actual and meaningful weight variations of the bin.

To do so, each experiment was regarded as a time series. This allowed us to seasonally decompose each series and therefore keep its *trend* dimension by discarding its *residual* and *seasonal* parts. The rationale for this decision was based on that the trend dimension actually captured most of the meaningful weight changes of the bin during each experiment.

### 3.5. Data Clustering

Once the sensor data were smoothed, it was possible to group them to represent different behavioral patterns related to plastic-waste generation in households. To do so, we first extracted five different features from each experiment’s time series, namely:

- The mean weight of the bin contents throughout the experiment (*m*).
- The value of the first quantile of the bin contents during the experiment ($q_{25}$).
- The value of the third quantile of the bin contents during the experiment ($q_{75}$).
- The number of usages of the bin during the experiment, i.e., the number of times the user puts one or more items in the bin (*u*).
- The number of items that the user actually places into the bin. This is calculated as an estimation based on the assumption that an average item weights around 10 g (*i*).

Please note that adding the quantile-based variables ($q_{25}$, $q_{75}$) might cause multicollinearity with respect to the mean weight variable *m*. However, both features have been included as part of the clustering step to provide a more descriptive representation of each cluster. This verbose representation made it easier to link each cluster’s centroid to a particular user profile as it will be described in Section 4.4.

The clustering algorithm *K-means* [28] was fed with these features to uncover different latent patterns in the experiments. Thus, each cluster was regarded as different user profile within the system’s contextual operation.

### 3.6. Web Application

One of the contributions of this work is the development of a web application that enables a continuous data visualization of the weight data for the plastic waste in the smart bin (see the dashboard component in Figure 1). This application also includes a module for predicting the generation of plastic waste in the smart bin (see Section 3.7).

The development of this application has followed the SCRUM methodology [29]. This methodology divides the application development into small stages, known as *sprints*, which offer a high degree of adaptation and flexibility and customer testing before finalizing the development. The SCRUM team was led by the Product Owner, role that corresponded to Andrés Muñoz, who stated the requirements of the applications as *user stories*. Fernando Terroso-Sáenz acted as the SCRUM Master, ensuring that the SCRUM practices and principles were followed in the project. Finally, the Development Team consisted of Navjot Sidhu and Alberto Pons-Buttazzo, who created and maintained the software. For this development, eight sprints, seven user stories, 28 tasks and 390 h were needed, resulting in an average of 14 h for each task.

For the web application, PHP was used for the back end, whereas the front-end was developed with HTML, Bootstrap and the JavaScript language. Bootstrap is a library for web applications that offers multiple tools to easily customize a website with free templates, a responsive design and maintaining browser compatibility. In addition, the following software tools were used: phpMyAdmin (MySQL administration), Visual Studio Code (source code editor), and XAMPP to manage the Apache web server and the database. The Python programming language was used for the predictor module.

### 3.7. Weight Prediction Method

Another key feature of the proposed system is the prediction of the weight of the plastic waste in each bin (see Figure 1). The three-step procedure applied to this end is explained next.

First, we identify the meaningful variations in the weight of the bin through an online analysis of the measurements from the scale. In brief, each new measurement $w_{i}$ reported by the scale at a time instant $t_{i}$ is considered a meaningful weight variation (MWV) $w_{i}^{m}$ if the following three conditions are fulfilled:

1. $w_{i}-w_{j}^{m}\geq \Delta_{m},j<i$
2. $t_{i}-t_{j}\geq \Delta_{t},j<i$
3. $w_{i}\geq w_{i+1}$

The first condition ensures that the difference between a new value $w_{i}$ and the most recent MWV $w_{j}^{m}$ is actually meaningful. The second one ensures that the time difference between such a previous $w_{j}^{m}$ and the new value is larger than a certain threshold $\Delta_{t}$. Please note that a time instant *t* in this setting refers to the time in seconds after the experiment under consideration began. The third condition ensures that the following weight value $w_{i+1}$ does not indicate a higher value than the current one, and therefore $w_{i}$ can be considered a MWV $w_{i}^{m}$. It should be noted that all the measurements come from the smooth version of the data collected from the weight sensor according to the data cleaning stage described in Section 3.4.

In the second step, each new meaningful variation $w_{i}^{m}$ is stored in a set W. This set comprises all the meaningful weight changes in the bin detected so far in an experiment. It is used to feed a linear-regression model whose goal is to predict the weight of the bin given a future time instant *t*, $w_{t}$, expressed as

$$w_{t}=\beta_{0}+\beta_{1}\times t$$

Please note that this model is re-trained each time W is enlarged with a new measurement.

In the third and final step, the liner regression model is used to estimate how long it will take to fill the bin. Assuming that the maximum weight of the bin is defined by $w_{max}$, such a time instant $t_{max}$ can be estimated from the original model as

$${\hat{t}}_{max}=\frac{w_{max}-\beta_{0}}{\beta_{1}}$$

Therefore, the time horizon to fill the bin will eventually be ${\hat{t}}_{max}-t_{now}$ where $t_{now}$ is the current time instant.

All in all, this approach defines a particular regression model for each experiment. In that sense, the complexity of the adopted model is quite low as it takes the form of a univariate linear regression. This will ease the actual scalability of the proposal by instantiating a large number of ad-hoc models without requiring an expensive computational infrastructure.

**Figure 4:**
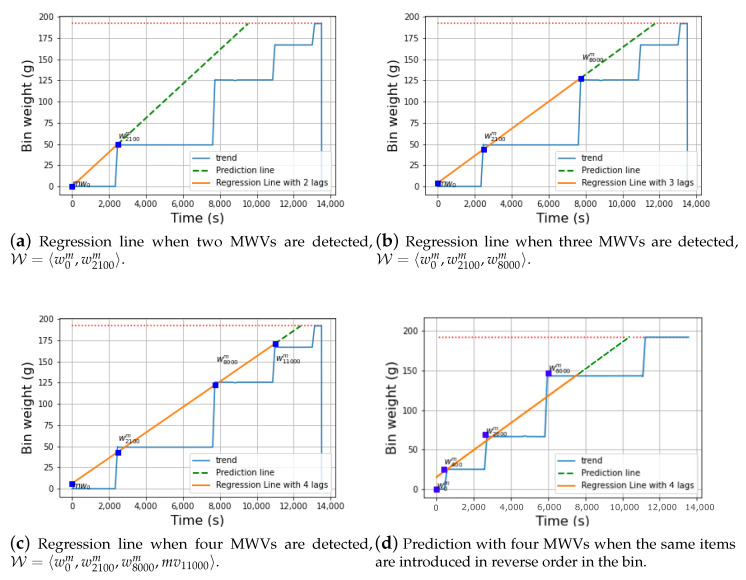
Example of the prediction mechanism for an experiment. The blue squares represent MWVs. The horizontal dotted red line indicates the maximum weight of the bin $w_{max}$.

For the sake of clarity, Figure 4 depicts an illustrative example of the aforementioned mechanism given three different time instants of a particular experiment. In the first moment (Figure 4a), two different MWVs have been identified at timestamps 0 and 2100. Therefore, the set W comprises such variations $m_{0}$ and $m_{2100}$ giving rise to a regression line whose projection is depicted as a dotted green line. As it can be observed, this projection line reaches the $w_{max}$ value (horizontal dotted red line in Figure 4a) at a time instant rather far to the actual one giving rise to a quite large prediction error (∼5000 s).

After that, a new MWV is detected at time instant 8000 which gives rise to a new linear-regression line based on the new set W (Figure 4b). With this new point, the regression line approximates better to the filling instant as its projection reaches the maximum weight closer to the actual time instant with an error below 2000 s. Finally, a fourth MWV ($mv_{11000}$) is added to compose a new regression line (Figure 4c). Again, it can be seen that the new projection can predict the filling instant even better than the other two regression lines with an error below 1000 s. Unsurprisingly, adding more MWVs to the model improves its prediction capabilities.

It is worth mentioning that changing the order of the items when they are introduced in the bin might slightly affect the accuracy of the predictor. However, the weight range of the plastic items that are introduced in the bin is quite limited because, for example, the weight difference between a small plastic bottle and a big one is within the range of a few grams. Consequently, the MWVs that give rise to the regression line usually involve similar weight increments. As a result, the order at which such increments occur does not meaningfully change the actual slope of the regression line and its associated prediction. Figure 4d shows the prediction of the model when the same plastic items are introduced in the bin in reverse order. As observed, the projection of the regression line reaches the $w_{max}$ level with a similar error than the one obtained with the original order.

### 3.8. Composition of the Routes for Waste-Pickers

The weight predictions described above feed the module in charge of composing the routes for the set of registered waste-pickers WP (see Figure 1). This module executes the following approach to create the set of routes for these waste-pickers.

First, each waste-picker wp∈WP is required to indicate the hours of the day $H_{wp}=\langle h_{wp}^{min},h_{wp}^{max}\rangle$ that he/she is available to perform a collection route.

Next, every day at the beginning of each time range $H_{wp}$, the system collects the set of pick-up points to be covered $P=\langle p_{1},p_{2},..,p_{N}\rangle$ where *N* is the total number of bins under control. Each pick-up point $p_{i}$ is defined by a tuple $\left(l_{i},{\hat{t}}_{max,i}\right)$ where $l_{i}$ is the location of the house for the *i*-th bin and ${\hat{t}}_{max,i}$ is the estimated filling hour returned by its associated predictor (Section 3.7).

Therefore, the definition of a personal collection route for a waste-picker wp∈WP can be formulated as the following problem:

**Given** a set of pick-up points P and an hour range $H_{wp}$, **Find** an ordered sequence $S_{wp}=\langle \left(p^{1},h^{1}\right)\to \left(p^{2},h^{2}\right)\to ...\to \left(p^{s},h^{s}\right)\rangle$ where each tuple $\left(p^{j},h^{j}\right)$ indicates that the pick-up point $p^{j}\in P$ should be visited at the $h^{j}$ hour of the day ($h^{j}<h^{k},\forall k>j$).

To obtain a set of suitable sequences, the following restrictions must apply in the generation process:

1. $h^{1}\geq h_{wp}^{min}$ and $h^{s}\leq h_{wp}^{max}$; and
2. $\forall (p^{j},h^{j})$, $h^{j}\leq {\hat{t}}_{max,j},j\in \left[1,s\right]$

The first condition ensures that the time duration of the calculated route fits into the hour range defined by the waste-picker. The second one ensures that the waste-picker will-collect the plastic waste at the *j*-th point of the sequence before its estimated filling hour (${\hat{t}}_{max,j}$).

It is worth mentioning that the set of pick-up points not included in a route $S_{wp}$ gives rise to a new set $P^{\prime }$ of pick-up points. This new set will be used to compose the route of other waste-picker by following the same approach. This process is repeated until no waste-pickers are left or all the bins are covered.

To conclude, note that this problem fits into the category of *vehicle routing problems with time windows* [30]. Thus, we have used the ILOG solver [31], a well-known constrained programming system, to generate the collection routes of the waste-pickers by considering the definitions and restrictions described above. In brief, this algorithm follows a tree-based search on the solution space to find routes that accomplish with all the listed restrictions.

## 4. Results

This section describes the main findings and results of the system proposed in Section 3. First, it is described the particular urban setting where our solution has been tested (Section 4.1) and the data collected for the simulation of the system functionality (Section 4.2). Next, we analyze the results of each module of the system in this urban setting (Section 4.3–Section 4.6).

### 4.1. Evaluation Setting

The performance of the proposed smart-collaborative plastic-waste management system has been simulated in the city of Quezon, Philippines. The Philippines as a country has not completely managed the plastic-waste problem yet. The country is plagued with drainage blockages due to plastics that usually occur during floods [32], posing a serious hazard to the population. Quezon City is the largest and most populous city in the Philippines, producing 262 tons of plastic waste per day. With 3,085,227 people and an area of 161.126 km${}^{2}$, multiple waste management plans have been tried and tested until today with different results [33], showing that there is still a need for ideas and projects to improve waste management.

With the aim of assisting the management of plastics through household smart bins for people with disabilities, senior citizens and COVID-19 affected residents, we have focused on data mainly about these profiles in the neighborhoods of Quezon City. In an Ecological Profile report made by the government office of Quezon City in 2015 [34], 34.92% of the total population are people with mental, speech, orthopedic, visual or hearing disabilities, among others. According to the report, the senior citizens are also included in this vulnerable group. In 2015, the estimated total number of people over 60 years old was 162,158.

In this study, three neighborhoods (known as *barangays*) of three different districts in Quezon are involved for a simulation scenario: Don Manuel under districts Galas, Commonwealth under the district with the same name and Mariana in New Manila. These neighborhoods represent a 0.13%, 6.75%, and 0.38% of the total population, respectively (https://www.philatlas.com/luzon/ncr/quezon-city.htmlsectionBrgys (accessed on 15 June 2021)). We have used this setting to allocate the different households simulated in the study and thus calculate the real distances for the route-planning algorithm described in Section 3.8.

In terms of socio-demographic profile of each neighborhood, in Don Manuel the computed age dependency ratio is 22 youth dependents to every 100 working age population. Commonwealth’s ratio is 43 and in Mariana it is 17 to every 100 working age population, respectively. Regarding the old-age-dependency ratio in these 3 neighborhoods, Mariana has the highest ratio of 11 to every 100. In terms of COVID-19, the city with the highest cases is Quezon City as of May 2021, with more than 150 active COVID-19 cases only in Barangay Commonwealth (https://quezoncity.gov.ph/covid19counts/qc-covid-19-update-as-of-may-11-2021-8am/(accessed on 15 June 2021)).

### 4.2. Data Collection

**Figure 5:**
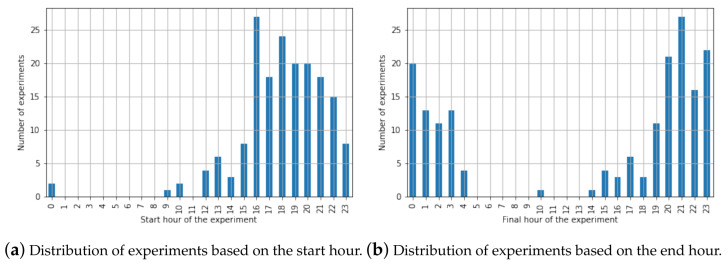
Distribution of the experiments based on their initial and end hour.

During the data collection stage explained in Section 3.3, 176 different experiments were performed from 19 October 2019 to 30 January 2021. The average time length of each experiment was 235.18 min with a standard deviation of 65.48 min. Figure 5 shows the distribution of the start and end hours of the experiments. As observed, most of the experiments were taken during afternoons and evenings.

### 4.3. Data Curation

**Figure 6:**
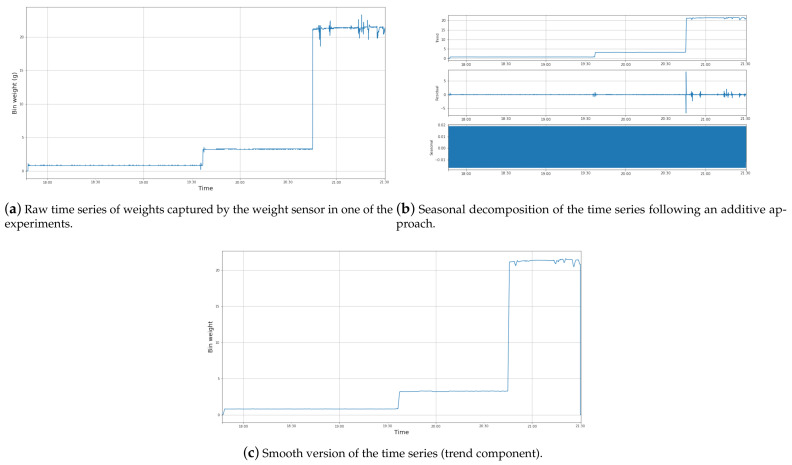
Data cleaning example.

As an illustrative example of the data filtering process described in Section 3.4, Figure 6a shows the time series of weights captured by the scale sensor during one of the experiments. As observed, the series is defined by a set of sharp weight increments. These moments indicate the time instant at which a user put one or more plastic items in the bin. Hence, the time series in Figure 6a reflects that the bin was used two times during the experiment since two sudden increments can be observed at different moments of the experiment. Apart from these increments, it is possible to observe some noisy points at the tail of the time series. This is because of the fluctuation mentioned in Section 3.4 given the high sensitivity of the weight sensor.

The seasonal decomposition of the time series is depicted in Figure 6b. As it can be seen, the time series is mainly defined by its *trend* dimension. This part of the time series indicates the clear evolution of the sensor values throughout the experiment, as it does not include the aforementioned noisy parts of raw values. Consequently, we were able to perform a lightweight filtering of this time series by just keeping its trend component (Figure 6c). Please note that this trend does not suffer from the noise observed in the raw time series.

Finally, the set of the 176 filtered time series were used for the next steps in the system, namely clustering and prediction.

### 4.4. Generated Clusters

**Figure 7:**
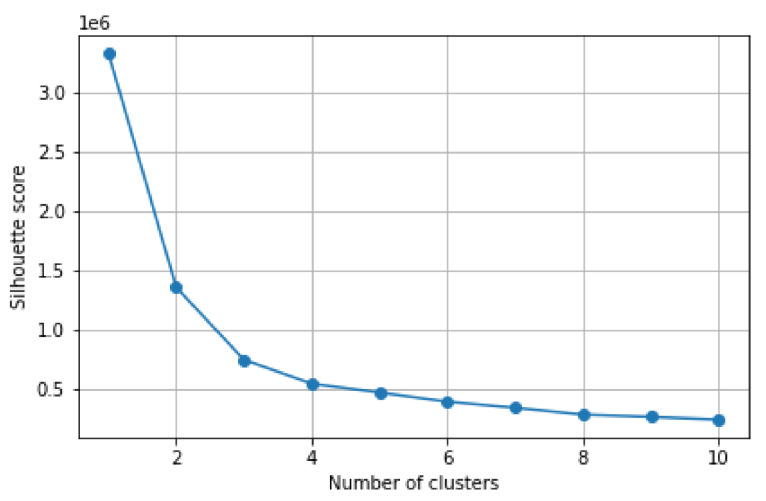
Silhouette score for different number of clusters (*k*).

Regarding the clusters obtained from the filtered time series and based on the features described in Section 3.5, Figure 7 shows the silhouette score of the K-means algorithm for different values of clusters *k*. As observed, the *elbow* point of the plot occurs when *k* is set to three. This indicates that such several clusters provides the best fit regarding the similarity of the entities within each cluster and the dissimilarity among different clusters. The centroids defining each cluster based on the five input features as explained in Section 3.5 are shown in Table 3.

In our simulation scenario, these centroids are representing a particular user profile in terms of generating plastic waste when using the smart bin. As a result, each centroid could be mapped to a particular behavior as follows:

- $c_{1}$: Family of two. One person with a disability who is working from home as an online teacher and another who works from home as a private money lender to small business owners. Both living in a one-bedroom flat in Barangay Don Manuel.
- $c_{2}$: Family of four. COVID-19 lockdown affected residents. One person is an essential worker, works as a jeepney/public transport driver, one person runs a small bakery business from home. Two kids who are studying from home. All living in a two-bedroom house in Barangay Commonwealth.
- $c_{3}$: Family of four with a caretaker. Husband and wife are working as doctor and nurse. Two kids who are in high school, and grandparents who stay at home. A caretaker comes to order/cook for the senior citizens. All living in a three-bedroom house in Barangay Mariana.

The rationale behind the assignment of these behaviors to each cluster is based on the actual information about these Barangays: Cluster $c_{1}$ in Don Manuel has one of the smallest areas in Quezon City with 0.238 km${}^{2}$. Additionally, the highest age group percentage is of 12.50% for 20 to 24 years old, and the generation of plastic waste is significantly low [35]. Cluster $c_{2}$ in Commonwealth has an area of 3.570 km${}^{2}$. Additionally, the age group of 15 to 64 years old have the highest distribution by percentage at 67.74% and the average daily plastic-waste production amounts to 330 cubic meters per day [36,37]. Finally, cluster $c_{3}$ in Mariana has an area of 1.664 km${}^{2}$. The age group with the highest percentage is 25 to 29 with 10.86 and the average daily plastic waste amounts to 1.65 cubic meters per day% [38,39].

### 4.5. Prediction Results

**Figure 8:**
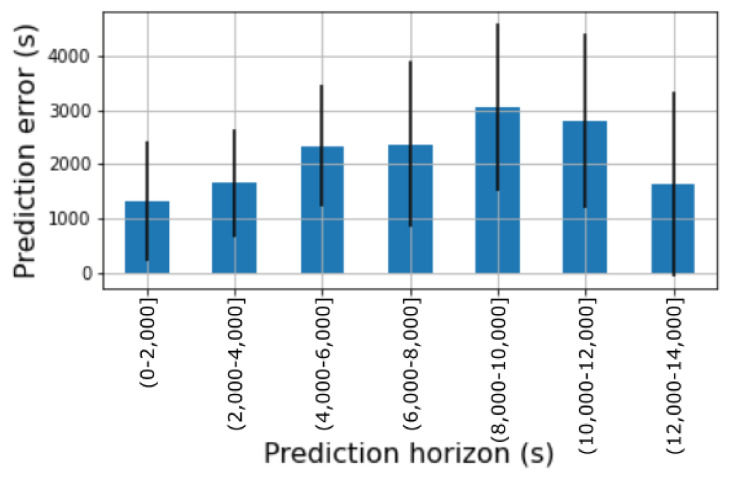
Residual error of the prediction model for different time horizons.

Given the predictor model in Section 3.7 aimed at forecasting the time when a bin is likely to be completely filled, we set $\Delta_{m}$ to 5 g and $\Delta_{t}$ to 200 s. This configuration was calculated by means of a grid search in the input space of both parameters. For this configuration, the solution had an average residual error of 2167 s (∼36 min) ±1390 s (∼23 min). Figure 8 shows the residual error ($|t_{max}-{\hat{t}}_{max}|$) of this model for different time horizons. For instance, for a time horizon below 2000 s (∼33 min) the error of the model was 1320 s (∼22 min) on average. It is observed that the accuracy of the model degrades as long as the prediction horizon increases.

However, a meaningful drop in the residual error occurs for very long time horizons between 12,000 and 14,000 s. The predictions for such horizons are provided by models fitted with W sets comprising the very first meaningful variations of an experiment (that is, the very first plastic items place inside the bin). This might suggest that the initial set of plastic items inserted in a bin might actually define the whole waste behavior of the target users at a high degree.

**Figure 9:**
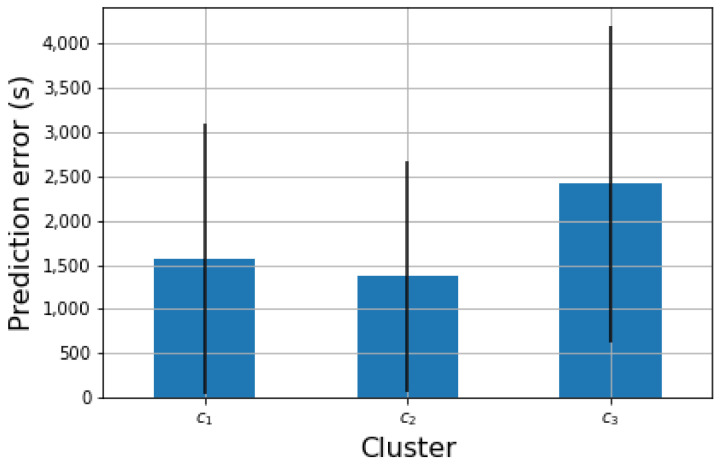
Residual error of the prediction mechanism given the identified clusters.

Figure 9 shows the distribution of the residual error for each of the user profiles defined in Section 4.4. It is observed that the accuracy of the predictor was lower for the experiments with cluster $c_{3}$, with a residual error of 3352 s (∼55 min) on average. For the other two clusters, the error was around 2000 s (∼33 min.).

From Figure 8 and Figure 9 it is observed that the predictor error ranges between 1200 and 4600 s (20 and 76 min). These predictions are used by the route generation algorithm to compute the pick-up hour for each bin as stated in Section 3.8. We believe that this error range is small enough to generate a pick-up hour close enough to the actual moment when the bin is full. In the worst case, users will have their bin completely full for around 76 min before a waste-picker arrives at their home. This seems a sensible time period to wait given the benefit that the system would bring to the user.

**Figure 10:**
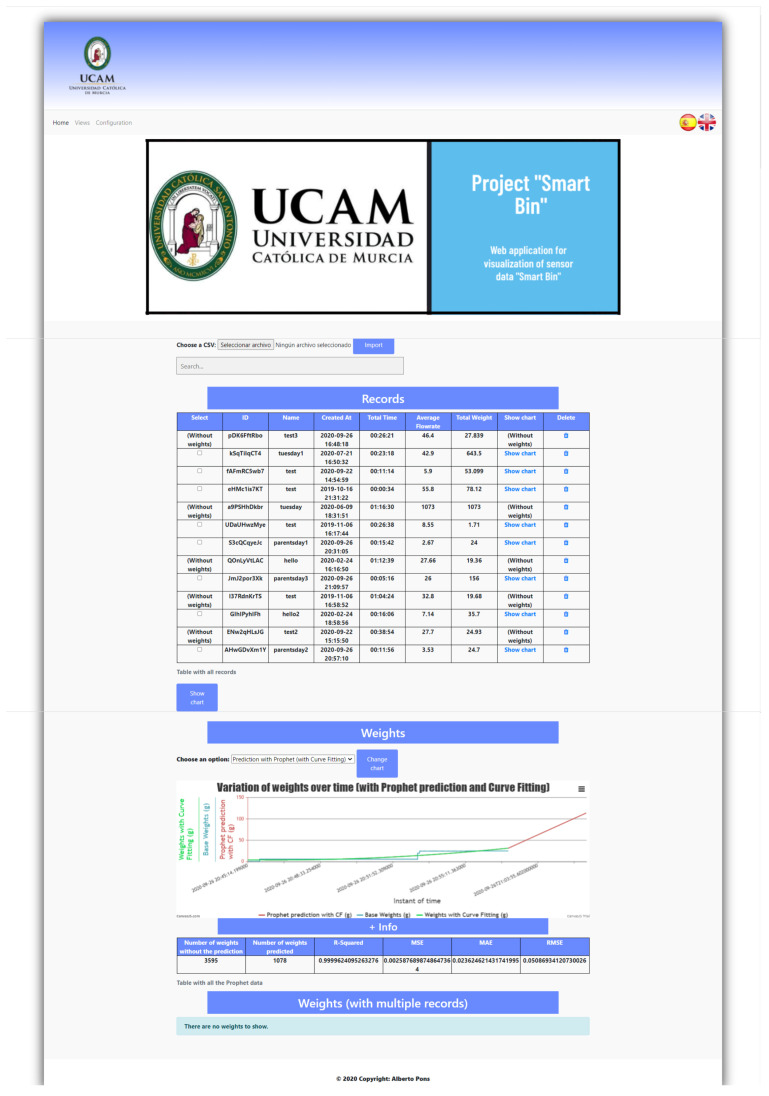
Main view of the web application. The upper section displays the list of experiments whereas the bottom one shows the time series of one of them.

Finally, the web application included in our proposal (Section 3.6) displays the time series of each experiment along with its associated prediction as shown in Figure 10. This allows the timely control and validation of the state of each bin in a real-world deployment.

### 4.6. Composition of the Collection Routes

The evaluation of the route generation algorithm explained in Section 3.8 has been performed in a simulated test-bed scenario within Quezon City for the clusters defined in Section 4.4.

**Figure 11:**
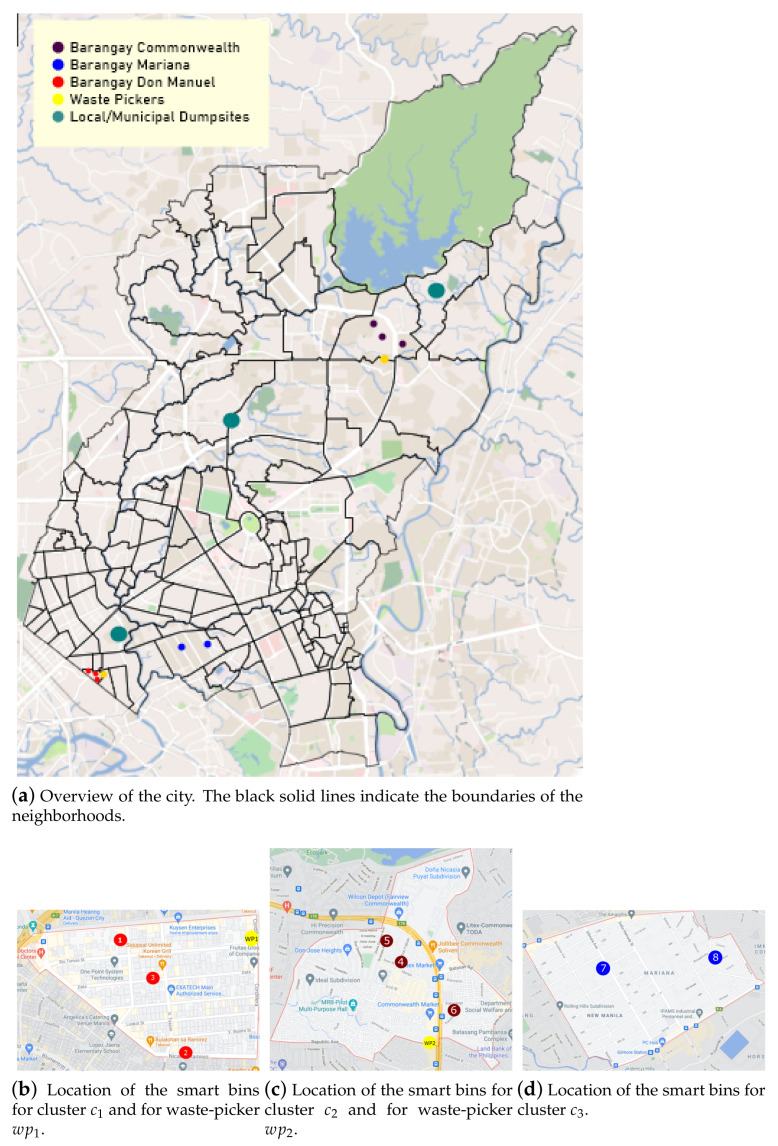
Quezon City map showing the location of the houses with smart bins and the waste-pickers’ homes. Each colored dot indicates a particular location. The red dots belong to cluster $c_{1}$, the dark red ones belong to cluster $c_{2}$ and the blue ones belong to $c_{3}$. The waste-picker houses are shown as yellow dots and the dumpsites are depicted as green dots.

To run the simulation, eight household smart bins and two waste-picker locations were defined within the city area. Then, each bin location was linked to a particular cluster for plastic-waste behavior. As shown in Figure 11, six smart bin locations in the south of the city were assigned to clusters $c_{1}$ (locations $l_{1}$, $l_{2}$, $l_{3}$) and $c_{2}$ ($l_{4}$, $l_{5}$, $l_{6}$), respectively, and two more in the north to $c_{3}$ ($l_{7}$, $l_{8}$). The set of waste-pickers was defined as $\mathrm{WP}=\langle wp_{1},wp_{2}\rangle$. Furthermore, we also considered the location of three municipal dumpsites at Quezon City. They are depicted as green dots in Figure 11a. Please note that these dumpsites are an important contextual factor to be considered. This is because waste-pickers need to deposit the plastic waste from the target houses in any of these dumpsites during their collection routes.

Once the scenario was set, each smart bin location $l_{i}$ was associated with a subset $E_{i}$ of the data collection experiments (out of 176) by considering its cluster (Section 4.2). For example, $E_{7}$ comprised experiments related to cluster $c_{3}$. In this manner we simulate a real-time behavior of the use of the household smart bins by means of an iterative approach. For each day in the study period (from 19 October 2019 to 30 January 2021 according to Section 4.2), we extracted a particular experiment related to that day, if any, from each set $E_{i}$. Next, for each experiment we kept its weight evolution of its first *k* items. A particular experiment gave rise to as many *sub-experiments* as its total number of items (see Table 4). Then, a prediction for the target *sub-experiment* of each location was generated giving rise to a set or pick-up points $P=\langle \left(l_{1},{\hat{t}}_{max,1}\right),..,\left(l_{n},{\hat{t}}_{max,n}\right)\rangle$ (n≤8). Moreover, the two waste-pickers had the following range of available hours, $H_{wp1}$ = (16:00, 23:00), $H_{wp2}$ = (20:00, 02:00). Consequently, the simulated real-time behavior can be regarded as a two-level loop, one moving across the days and a nested one moving across the number of items of the experiments.

The set of pick-up points along with the range hours of the waste-pickers fed the ILOG solver to compose the required collecting routes. To do so, we made use of the implementation provided by the *OR-tools* suite [40], open-source software for optimization.

This suite requires the time distances among the target locations to compose the final routes. To study the impact of the means of transport used by the waste-pickers to cover the routes, we defined distance matrices based on three means of transport, namely bike, car and on foot. The travel times were calculated by means of the *Google Maps* web service (https://www.google.com/maps (accessed on 24 May 2021)). For completeness, Appendix A shows the travel time matrices for each means of transport (see Table A1 for bike, Table A2 for on foot and Table A3 for car). Furthermore, we also considered that the number of plastic-waste bags that a waste-picker can carry at the same time depends on the specific means of transport. It was assumed that a waste-picker can carry only two bags at the same time when moving on foot or by bike and eight when moving by car. This was done by forcing the route composer to include a visit to the closest dumpsite when such several bags are reached.

For each route, its *collection rate* was calculated. This metric indicates the percentage of bins included in the route that would have been eventually collected by the waste-picker before they were filled. This is possible to calculate because each route comprises the collection hour for each bin and the actual filling hour of the bin, which is available through the original experiment.

**Figure 12:**
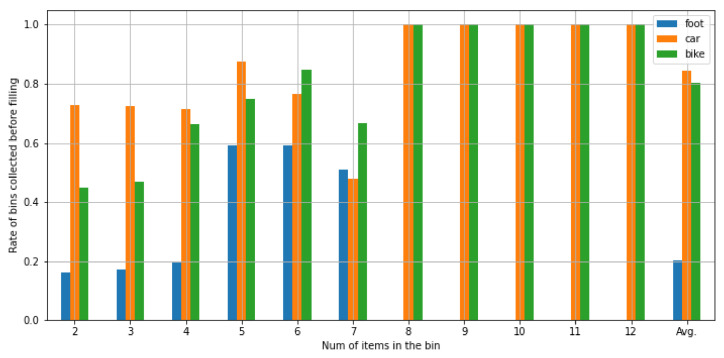
Collection rate per means of transport and number of items in the bin.

Figure 12 shows the collection rate for the three means of transport. This rate is shown taking into account the number of items that were already inserted in the bin when the route composition algorithm was performed. For example, the leftmost blue column shows that the average collection rate for routes based on predictions generated when there were two plastic items in the bin and the waste-pickers moved on foot was 0.18 (i.e., 18% of the bins were collected before they were filled).

According to Figure 12, the solution achieved a collection rate around 0.8 on average when bikes or cars were used as means of transport. However, this rate remarkably dropped when waste-pickers moved on foot (see the last *Avg.* column in Figure 12). This is because this means of transport required very long walks to the dumpsites, requiring an extra time with respect to the other means of transport.

Another interesting finding is that the collection rate when using bikes and cars increases as long as the predictions are based on a larger number of items. As a matter of fact, their rate equals to 1 (i.e., all the bins are collected before they are actually filled) when the predictions used by the ILOG solver rely on 8 or more items regardless of the means of transport. The reason of this behavior is that the predictions based on a low number of items have a larger variability than those based on a high number. This high variability makes the route composer to receive as input pick-up points with very different filling hours, and therefore it is not able to find a suitable route covering all the locations in most situations. However, if the estimated filling hours rely on predictions based on a larger number of items, they tend to be more homogeneous covering a smaller range of hours within a day. This makes it easier for the solver to find a feasible pick-up sequence. Regarding the predictions based on a lower number of items, the rate is usually higher when the waste-pickers use car or bikes.

Regarding the results when waste-pickers move on foot, it is observed a different behavior than for the other two means of transport. Although the system achieved collection rates from 0.18 to 0.59 when predictions were based on seven or less items (see Figure 12), it was not able to compose collection routes when a larger number of items were included in the prediction step. This is strongly related to the homogeneity of the predictions explained in the previous paragraph. Since the range of hours is smaller in this case, the route composer is not able to find a route that covers all the houses along with the required visits to the dumpsites.

**Figure 13:**
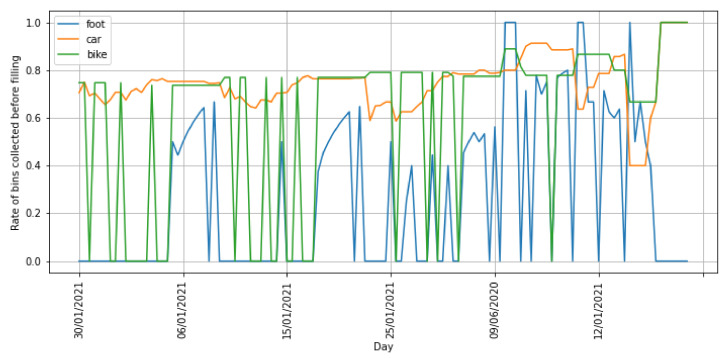
Collection rate per means of transport and day.

Furthermore, Figure 13 shows the collection rate for each of the days under study. As observed, the rate fluctuations for the routes covered on foot and by bike were higher than for the routes covered by car. Those walking and biking routes had a rate of 0 in several days indicating that it was not possible to compose a route able to visit any of the bins’ houses before the filling hour. However, the car-based routes exhibited a higher stability with rates above 0.7 in most of the days.

As an illustrative example of a specific route, given the following pick-up points $P=\langle \left(l_{1},20\right),\left(l_{2},20\right),\left(l_{3},19\right),\left(l_{4},23\right),\left(l_{5},22\right),\left(l_{6},21\right),\left(l_{7},18\right)\rangle$, the route solver is able to compose the following route for $wp_{1}$ when she moves by bike,

$S_{wp1}=\langle \left(l_{7},18\right)\to \left(l_{3},19\right)\to (DS_{2},19)\to \left(l_{1},20\right)\to \left(l_{2},20\right)\to (DS_{2},20)\to \left(l_{6},21\right)\to \left(l_{5},22\right)\to (DS_{3},23)\to \left(l_{4},23\right)\rangle$

As a result, the waste-picker would be able to reach bins at $l_{1}$ and $l_{2}$ and leave their bags at dumpsite $DS_{2}$ during the same hour range, since it only takes 4 min to go from $l_{1}$ to $l_{2}$ and 15 min to go from $l_{2}$ to $DS_{2}$ according to the time matrix for bike routes (Table A1). The same occurs for $l_{4}$ and $DS_{3}$ because it takes 49 min to move from one location to the other.

## 5. Discussion

This work falls into the research line on the application of smart and assistive technologies to waste management. Although most works in this area focus on municipal waste management through different types of algorithms and smart bins deployed in the streets of a city, this study proposes a more personalized management of plastic waste in the home environment. Hence, the aim is to offer a more appropriate service to certain groups of citizens, such as the elderly or people with reduced mobility, to help them in their day-to-day waste disposal.

There are some works that propose a certain degree of collaboration for waste management using gamification as a collaborative tool [19,41]. However, they do not offer an intelligent collaborative collection service such as the one proposed in our work. Therefore, the novelty of our proposal lies in the integrated management of plastic-waste monitoring and the planning of optimal routes for its collection.

Regarding the limitation of this work, we have encountered some challenges in operational terms. Thus, the proposed infrastructure only relies on the evolution of the weight of the bin contents for the clustering and prediction tasks. It is possible that monitoring the actual number of items within the bin would allow for a more robust system. In this sense, ultrasonic sensors could be used as an alternative to perform this task. However, adding this sensor to our infrastructure will inevitably increase its cost. Since one of our goals is the deployment of the system in developing countries, providing a cost-effective solution is a paramount requirement.

Furthermore, our system also assumes that the target bins are always available to be collected by the waste-pickers. Although this may represent a limitation in the use of the system, we believe it is a sensible assumption for two reasons. First, the bins are located at the users’ homes, so they are in a controlled environment. Secondly, the system is mainly aimed at elderly or people with reduced mobility who are quite restrained when it comes to leave their homes. Moreover, it would be possible to develop an early warning system to notify users about the expected pick-up time according to the planned route returned by the scheduler. At the same time, it would be possible to improve the system by allowing waste-pickers to postpone part of their routes to the next day if the expected time of arrival to certain houses is too late at night.

As for the methodological approach, the SCRUM methodology has proved to be successful for the development of this work. Nonetheless, we have found some limitations in the design of the experiments for data collection. It would have been desirable to use bins with different sizes to achieve more realistic scenarios, as well as a more heterogeneous identification of user profiles. However, the results obtained with the proposed evaluation settings allows us to validate our proposal to manage plastic household waste efficiently by means of a low-cost smart bin.

Finally, it is worth mentioning the implications and applicability of the proposed system, both at small and large-scale. Regarding the waste collection system of the Philippines, it consists of waste-pickers and garbage trucks that collect waste using a door-to-door approach. However, this is a fixed-route service without taking into account the needs of certain groups of citizens or the actual state of the bins. Hence, our application may help organize this approach in a more efficient and personalized manner. At the same time, and in collaboration with municipal authorities, our system may help in a more sustainable waste management if extended to the entire population, as it may prevent citizens from uncontrolled dumping of waste on the streets by encouraging them to use the collaborative application. Our proposal can be also extended to other cities in developing countries suffering the same waste-management problem with plastics or other recyclable items such as glass or paper.

## 6. Conclusions and Future Work

Problems related to plastic-waste generation and collection have been long ongoing, especially in countries without or limited waste-management policies. Furthermore, plastic production has significantly increased during the COVID-19 pandemic. Disposable products such as masks, PPE kits, and gloves have doubled the plastic waste, which makes communities and countries in need of new and creative waste-management solutions. Additionally, citizens with disabilities, seniors and those in COVID-19 quarantine may have difficulties throwing the plastic waste in a daily basis.

This paper proposes a collaborative solution for household plastic-waste collection focused on the aforementioned groups. This solution is composed of a simple smart bin equipped with a high-sensitivity weight sensor that feeds a predictor model to forecast the amount of plastic waste in each bin. The results from the predictor model are forwarded to a route-planning algorithm to generate optimum routes for waste-pickers or residential garbage collectors that allows them to maximize the number of bins collected in the shortest time. This proposal has been validated through a simulation in Quezon City (the Philippines) by identifying three user profiles related to plastic-waste generation in eight different locations and with the collaboration of two waste-pickers using 3 different means of transport. The collection rate for this simulation resulted in an average of above 0.8, i.e., more than the 80% of the household bins were collected before they were completely filled.

A future line of work is to apply our proposal to more diverse households and communities to identify more real and accurate profiles of plastic-waste generation. Another planned work is the use of real-time route matrices that include traffic conditions to improve the accuracy and efficiency of the collection routes.

## Figures and Tables

**Table 1:** Demographic characteristics of the participants of the experiments.

Type of Household	Age Group	Gender	Number of Experiments
one-person	24–33	female	76
two-persons	49–55	male, female	26
five people	18–24	male	58
workplace	24–38	male, female	16

**Table 2:** Data samples collected from the experiments with the household bins.

Date/Time	Total Time (S)	Total Weight (g)	Weight Data (g)
03/02/2021 20: 12	2525	207.8	0.00; 0.00; 0.00; 0.00; 0.50; 1.40; 3.80;
			63.70; 94.70; 91.80; 37.3; 21.3; 43.70;
			110.2; 110.2; 110.2; 110.2; 110.2; 110.2;
			110.2; 110.2; 110.2; 110.2; 110.2...;
02/02/2021 18:15	1013	112.5	0.00; 0.00; 0.00; 0.00; 0.00; 0.00; 0.00;
			0.00; 0.00; 0.00; 0.00; 0.70; 0.70; 2.20;
			76.90; 76.90; 76.90; 76.90; 76.90; 76.90;
			101.90; 101.90; 101.90; 101.90; 101.90;

**Table 3:** Values of the descriptive features for the three clusters extracted from the experiments.

Cluster	*m*	$q_{25}$	$q_{75}$	*u*	*i*
$c_{1}$	47.37	22.38	72.39	2.45	8.77
$c_{2}$	129.64	74.83	188.94	4.09	21.51
$c_{3}$	274.40	188.86	398.44	4.38	38.92

**Table 4:** Number of sub-experiments for each location used across the simulations.

Location	Num. of Sub-Experiments
$l_{1}$	369
$l_{2}$	233
$l_{3}$	151
$l_{4}$	379
$l_{5}$	217
$l_{6}$	109
$l_{7}$	223
$l_{8}$	124

## Data Availability

The source code of the project is available at https://github.com/fterroso/waste-collection-routes (accessed on 15 June 2021).

## Appendix A. Time Distance Matrices in the Experiments

This section contains the matrices describing the travel time among the locations in the simulation scenario.

**Table A1:** Time distance matrix based on bike displacements. A cell $c_{ij}$ indicates the time required to move from location *i* to *j* in hours:minutes:seconds format.

	${\mathrm{wp}}_{1}$	${\mathrm{wp}}_{2}$	$l_{1}$	$l_{2}$	$l_{3}$	$l_{4}$	$l_{5}$	$l_{6}$	$l_{7}$	$l_{8}$	${\mathrm{DS}}_{1}$	${\mathrm{DS}}_{2}$	${\mathrm{DS}}_{3}$
$wp_{1}$	0:00:00	1:06:00	0:04:00	0:05:00	0:03:00	1:12:00	1:14:00	1:05:00	0:12:00	0:13:00	1:30:00	0:10:00	2:10:00
$wp_{2}$	1:06:00	0:00:00	1:06:00	1:12:00	1:07:00	0:10:00	0:12:00	0:05:00	1:06:00	2:12:00	1:30:00	1:13:00	0:25:00
$l_{1}$	0:04:00	1:06:00	0:00:00	0:04:00	0:01:00	1:47:00	1:44:00	1:55:00	0:15:00	0:23:00	1:26:00	0:10:00	2:23:00
$l_{2}$	0:05:00	1:12:00	0:04:00	0:00:00	0:03:00	1:15:00	1:15:00	1:08:00	0:13:00	0:25:00	1:30:00	0:15:00	2:10:00
$l_{3}$	0:03:00	1:07:00	0:01:00	0:03:00	0:00:00	1:10:00	1:12:00	1:03:00	0:15:00	0:17:00	0:54:00	0:15:00	1:13:00
$l_{4}$	1:12:00	0:10:00	1:47:00	1:15:00	1:10:00	0:00:00	0:10:00	0:13:00	1:14:00	1:01:00	1:26:00	1:19:00	0:49:00
$l_{5}$	1:14:00	0:12:00	1:44:00	1:15:00	1:12:00	0:10:00	0:00:00	0:15:00	1:17:00	1:02:00	1:18:00	2:51:00	0:33:00
$l_{6}$	1:05:00	0:05:00	1:55:00	1:08:00	1:03:00	0:13:00	0:15:00	0:00:00	1:08:00	0:55:00	1:34:00	1:12:00	0:28:00
$l_{7}$	0:12:00	1:06:00	0:15:00	0:13:00	0:15:00	1:14:00	1:17:00	1:08:00	0:00:00	0:06:00	1:37:00	0:20:00	1:15:00
$l_{8}$	0:13:00	2:12:00	0:23:00	0:25:00	0:17:00	1:01:00	1:02:00	0:55:00	0:06:00	0:00:00	1:05:00	0:40:00	1:40:00
$DS_{1}$	1:30:00	1:30:00	1:26:00	1:30:00	0:54:00	1:26:00	1:18:00	1:34:00	1:37:00	1:05:00	0:00:00	1:30:00	1:40:00
$DS_{2}$	0:10:00	1:13:00	0:10:00	0:15:00	0:15:00	1:19:00	2:51:00	1:12:00	0:20:00	0:40:00	1:30:00	0:00:00	2:30:00
$DS_{3}$	2:10:00	0:25:00	2:23:00	2:10:00	1:13:00	0:49:00	0:33:00	0:28:00	1:15:00	1:40:00	1:40:00	2:30:00	0:00:00

**Table A2:** Time distance matrix based on walking displacements. A cell $c_{ij}$ indicates the time required to move from location *i* to *j* in hours:minutes:seconds format.

	${\mathrm{wp}}_{1}$	${\mathrm{wp}}_{2}$	$l_{1}$	$l_{2}$	$l_{3}$	$l_{4}$	$l_{5}$	$l_{6}$	$l_{7}$	$l_{8}$	${\mathrm{DS}}_{1}$	${\mathrm{DS}}_{2}$	${\mathrm{DS}}_{3}$
$wp_{1}$	0:00:00	2:43:00	0:06:00	0:06:00	0:06:00	2:57:00	3:06:00	2:48:00	0:35:00	0:49:00	1:54:00	0:13:00	3:15:00
$wp_{2}$	2:43:00	0:00:00	2:39:00	2:43:00	2:40:00	0:17:00	0:25:00	0:09:00	2:19:00	1:14:00	1:23:00	2:29:00	0:34:00
$l_{1}$	0:06:00	2:39:00	0:00:00	0:08:00	0:02:00	2:59:00	3:08:00	2:50:00	0:40:00	0:54:00	1:56:00	0:18:00	3:17:00
$l_{2}$	0:06:00	2:43:00	0:08:00	0:00:00	0:07:00	3:04:00	3:12:00	2:54:00	0:41:00	1:02:00	2:00:00	0:19:00	3:10:00
$l_{3}$	0:06:00	2:40:00	0:02:00	0:07:00	0:00:00	3:00:00	3:10:00	2:51:00	0:40:00	0:52:00	1:57:00	0:19:00	1:43:00
$l_{4}$	2:57:00	0:17:00	2:59:00	3:04:00	3:00:00	0:00:00	0:14:00	0:16:00	2:31:00	2:27:00	1:38:00	2:43:00	1:37:00
$l_{5}$	3:06:00	0:25:00	3:08:00	3:12:00	3:10:00	0:14:00	0:00:00	0:21:00	2:43:00	2:34:00	1:34:00	1:20:00	0:45:00
$l_{6}$	2:48:00	0:09:00	2:50:00	2:54:00	2:51:00	0:16:00	0:21:00	0:00:00	2:23:00	2:17:00	1:15:00	2:34:00	0:15:00
$l_{7}$	0:35:00	2:19:00	0:40:00	0:41:00	0:40:00	2:31:00	2:43:00	2:23:00	0:00:00	0:16:00	1:19:00	0:27:00	2:56:00
$l_{8}$	0:49:00	1:14:00	0:54:00	1:02:00	0:52:00	2:27:00	2:34:00	2:17:00	0:16:00	0:00:00	1:31:00	1:02:00	2:49:00
$DS_{1}$	1:54:00	1:23:00	1:56:00	2:00:00	1:57:00	1:38:00	1:34:00	1:15:00	1:19:00	1:31:00	0:00:00	1:42:00	1:59:00
$DS_{2}$	0:13:00	2:29:00	0:18:00	0:19:00	0:19:00	2:43:00	1:20:00	2:34:00	0:27:00	1:02:00	1:42:00	0:00:00	3:06:00
$DS_{3}$	3:15:00	0:34:00	3:17:00	3:10:00	1:43:00	1:37:00	0:45:00	0:15:00	2:56:00	2:49:00	1:59:00	3:06:00	0:00:00

**Table A3:** Time distance matrix based on car displacements. A cell $c_{ij}$ indicates the time required to move from location *i* to *j* in hours:minutes:seconds format.

	${\mathrm{wp}}_{1}$	${\mathrm{wp}}_{2}$	$l_{1}$	$l_{2}$	$l_{3}$	$l_{4}$	$l_{5}$	$l_{6}$	$l_{7}$	$l_{8}$	${\mathrm{DS}}_{1}$	${\mathrm{DS}}_{2}$	${\mathrm{DS}}_{3}$
$wp_{1}$	0:00:00	0:22:00	0:01:00	0:01:00	0:02:00	0:22:00	0:22:00	0:15:00	0:06:00	0:09:00	0:24:00	0:05:00	0:35:00
$wp_{2}$	0:22:00	0:00:00	0:19:00	0:20:00	0:18:00	0:06:00	0:05:00	0:04:00	0:17:00	0:18:00	0:13:00	0:23:00	0:11:00
$l_{1}$	0:01:00	0:19:00	0:00:00	0:02:00	0:01:00	0:32:00	0:21:00	0:18:00	0:10:00	0:20:00	0:25:00	0:07:00	0:35:00
$l_{2}$	0:01:00	0:20:00	0:02:00	0:00:00	0:02:00	0:35:00	0:35:00	0:30:00	0:06:00	0:20:00	0:27:00	0:07:00	0:35:00
$l_{3}$	0:02:00	0:18:00	0:01:00	0:02:00	0:00:00	0:35:00	0:40:00	0:20:00	0:07:00	0:09:00	0:27:00	0:07:00	0:24:00
$l_{4}$	0:22:00	0:06:00	0:32:00	0:35:00	0:35:00	0:00:00	0:08:00	0:09:00	0:55:00	0:50:00	0:16:00	0:26:00	0:14:00
$l_{5}$	0:22:00	0:05:00	0:21:00	0:35:00	0:40:00	0:08:00	0:00:00	0:06:00	0:55:00	0:50:00	0:17:00	0:28:00	0:16:00
$l_{6}$	0:15:00	0:04:00	0:18:00	0:30:00	0:20:00	0:09:00	0:06:00	0:00:00	0:50:00	0:43:00	0:19:00	0:29:00	0:10:00
$l_{7}$	0:06:00	0:17:00	0:10:00	0:06:00	0:07:00	0:55:00	0:55:00	0:50:00	0:00:00	0:04:00	0:24:00	0:09:00	0:35:00
$l_{8}$	0:09:00	0:18:00	0:20:00	0:20:00	0:09:00	0:50:00	0:50:00	0:43:00	0:04:00	0:00:00	0:23:00	0:11:00	0:34:00
$DS_{1}$	0:24:00	0:13:00	0:25:00	0:27:00	0:27:00	0:16:00	0:17:00	0:19:00	0:24:00	0:23:00	0:00:00	0:22:00	0:29:00
$DS_{2}$	0:05:00	0:23:00	0:07:00	0:07:00	0:07:00	0:26:00	0:28:00	0:29:00	0:09:00	0:11:00	0:22:00	0:00:00	0:38:00
$DS_{3}$	0:35:00	0:11:00	0:35:00	0:35:00	0:24:00	0:14:00	0:16:00	0:10:00	0:35:00	0:34:00	0:29:00	0:38:00	0:00:00
